# Shrews as Reservoir Hosts of Borna Disease Virus

**DOI:** 10.3201/eid1204.051418

**Published:** 2006-04

**Authors:** Monika Hilbe, Romana Herrsche, Jolanta Kolodziejek, Norbert Nowotny, Kati Zlinszky, Felix Ehrensperger

**Affiliations:** * University of Zurich, Zurich, Switzerland;; † University of Veterinary Medicine, Vienna, Austria;; ‡ United Arab Emirates University, Al Ain, United Arab Emirates

**Keywords:** Borna disease, Borna disease virus, virus reservoir, bicolored white-toothed shrew, *Crocidura leucodon*, Switzerland, immunohistochemistry, TaqMan real-time RT-PCR, RT-PCR, sequencing

## Abstract

Borna disease virus (BDV) is the causative agent of severe T-cell–mediated meningoencephalitis in horses, sheep, and other animal species in central Europe. Here we report the first unequivocal detection of a BDV reservoir species, the bicolored white-toothed shrew, *Crocidura leucodon*, in an area in Switzerland with endemic Borna disease.

Borna disease (BD) is a severe immunopathologic disorder of the central nervous system induced by infection with Borna disease virus (BDV), the prototype of a new virus family, *Bornaviridae*, within the order Mononegavirales. With 1 notable exception (strain No/98 [1,2]), all BDV isolates exhibit a highly conserved genome (*3*). BD primarily affects horses and sheep, although many animals can be infected experimentally (*4**–**6*). BD is restricted to well-defined disease-endemic regions in central Europe (*7*). The precise pathogenesis and epidemiology of natural BDV infections are unknown; however, several unique epidemiologic features point towards the existence of BDV reservoir populations other than the final hosts (*4**,**8**,**9*).

Laboratory diagnosis of BD relies on postmortem examination of the brain by histologic techniques, immunohistologic (IHC) testing, reverse transcription–polymerase chain reaction (RT-PCR), and recently TaqMan real-time RT-PCR (*5**,**7*). Histologically, BD manifests as mononuclear inflammation (meningoencephalitis), especially in the hippocampal area, where frequently intranuclear, eosinophilic (so-called Joest-Degen) inclusion bodies can be observed (*7**,**10*). The nucleoprotein (p38/40, open reading frame [ORF] I) and the phosphoprotein (p24, ORF II) are the 2 most important BDV target proteins and genes for IHC, RT-PCR, and TaqMan real-time RT-PCR, respectively (*4**,**5**,**7**,**8**,**10**–**12*).

## The Study

The objective of this study was to search for the putative natural reservoir hosts or vectors of BDV in an environment in which BD is endemic in horses and sheep. Eight moles, 3 shrews, and 87 mice of different species were trapped between 1999 and 2003 in a small village near Chur, Switzerland (Malix, located 1130 m above sea level), an area in which BD is endemic in horses and sheep. The animals were euthanized and stored at –20°C for later examination. Their brains were divided into 2 equal parts, one half was fixed in 4% formaldehyde, cut transversally into several equal parts and embedded in paraffin for microscopic evaluation; the other half was stored in tubes at –20°C. IHC was performed as described previously (*5**,**7**,**10*). A recently established TaqMan real-time RT-PCR system (Applied Biosystems, Rotkreuz, Switzerland) (*5*) was used to detect and quantify BDV nucleic acid in all brain samples and selected heart samples from the mice, shrews, and moles.

All samples that were positive by TaqMan real-time RT-PCR in the Zurich laboratory as well as selected negative samples were reevaluated blind in the Vienna laboratory by conventional RT-PCR, beginning with frozen parallel samples that had been stored. RT-PCR and sequencing were carried out as described by Kolodziejek et al. (*3*). The resulting amplicons of 1 shrew (no. 144) were sequenced by employing the ABI PRISM BigDye Terminator Cycle Sequencing Ready Reaction Kit (PE Applied Biosystems, Foster City, CA, USA) and an ABI Prism 310 genetic analyzer (PE Applied Biosystems).

Histologic examination showed no inflammation or degenerative processes in any of the 98 brains. Three of the 98 brains, however, were positive for BDV antigen by IHC with both monoclonal antibodies. Labeling of inclusion bodies and some intracytoplasmic staining was notable from the forebrain (prosencephalon) to the mesencephalon (Figure, panel A). The 3 BDV-antigen–positive brains originated from the 3 shrews investigated, while all samples from moles and different species of mice proved negative. Identical results were obtained by TaqMan real-time RT-PCR; all 95 mouse and mole brains were negative, while the brain samples of all 3 shrews were positive (Table).

**Figure:**
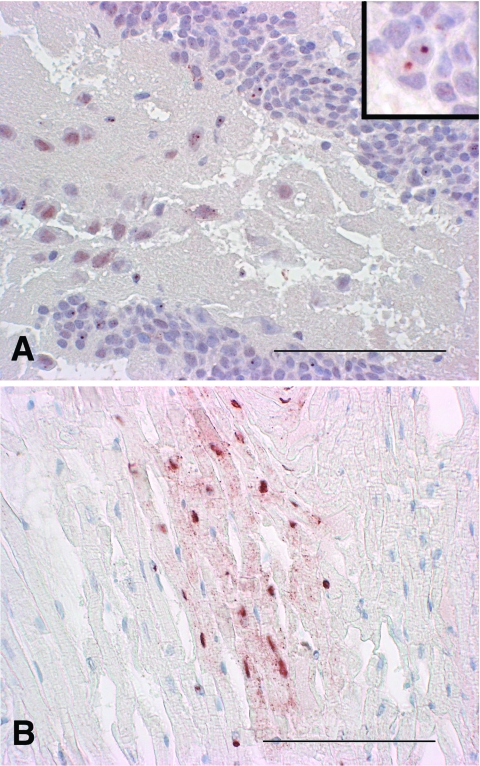
A) Distribution of Borna disease virus (BDV) p24 antigen in the hippocampus of shrew 144. Note the so-called Joest-Degen intranuclear inclusion bodies in multiple neurons in the gyrus dentatus; in some neurons, a homogenous intracytoplasmic staining can be seen (immunohistochemistry, ChemMate method [DAKO, Cygomation, Zug, Switzerland], ×40 [bar, 100 μm]). Inset: Joest-Degen intranuclear inclusion bodies are visible in multiple neurons in the gyrus dentatus (ChemMate method, ×100). B) Focal distribution of BDV p38/40 antigen in the heart of shrew 144: granular intracytoplasmic and homogenous intranuclear labeling (ChemMate method, ×40 [bar, 100 μm]).

**Table:** Summary of immunohistologic and real-time RT-PCR findings*

Case no.	Organ	Immunohistology	Real-time RT-PCR Ct values† p24 and p40 (mean of 2 analyses)	Real-time RT-PCR Ct values 18S rRNA (mean value)	Calibrated values‡ (virus copies; mean of 2 analyses)
134	Brain	p24 positive	p24: 14.93 / 17.53	13.34	p24: 271.9
		p40 positive	p40: 15.87 / 19.35	19.64	p40: 40.87
	Heart	p24 negative	p24: 28.07 / 29.54		p24: 2.866
		p40 negative	p40: 27.94 / 30.05		p40: 1.749
137	Brain	p24 positive	p24: 24.00 / 25.04	24.41	p24: 13,246
		p40 positive	p40: 25.50 / 26.95		p40: 2,218.8
	Heart	p24 negative	p24: 33.88 / 38.07	19.66	p24: 0.0204
		p40 negative	p40: 33.84 / 35.24		p40: 0.0366
144	Brain	p24 positive	p24: 16.27 / 17.44	13.98	p24: 216.04
		p40 positive	p40: 17.09 / 18.88		p40: 46.289
	Heart	p24 positive	p24: 32.32 / 33.92	23.83	p24: 2.8235
		p40 positive	p40: 31.68 / 35.17		p40: 1.3375

*Histologic findings: no lesions on brain or heart for all 3 cases. †RT-PCR, reverse transcription–polymerase chain reaction; Ct, threshold cycle no. ‡The calibrated values were calculated by using the sum of both normalized Ct-values of Borna disease virus p24 and p40, respectively, and dividing it by the normalized value of the 18s rRNA (*5*). Each organ was analyzed twice, and the evaluation was performed in duplicate. Please note that the viral load in each animal can be quite variable.

In addition to brain tissues, the hearts of the 3 BDV-positive shrews and of 2 BDV-negative mice were examined. In the heart of shrew 144, BDV-positive labeling was found in a multifocal pattern (Figure, panel B). The myocardiocytes showed intracytoplasmic and intranuclear labeling, but no inclusion bodies could be recognized. The hearts of the other 2 shrews and the 2 mice were negative by IHC. By TaqMan real-time RT-PCR, however, the hearts of all 3 shrews proved positive (Table), while the hearts of the mice were negative. The observed differences in the results obtained by the 2 methods can be explained by the higher sensitivity of the TaqMan method compared to IHC.

When the brains of the shrews were analyzed by conventional RT-PCR, all 6 assays yielded amplicons of the expected sizes in all 3 shrews, whereas mouse samples negative by TaqMan real-time RT-PCR were also negative by conventional RT-PCR. The compiled sequences of shrew 144 resulted in a stretch of 2,041 nucleotides (nt), composing the complete N, X, and P protein-encoding regions, as well as the 5′-untranslated region of the X/P transcript of the BDV genome. The shrew-derived sequence (GenBank accession no. DQ251041) was verified as the expected BDV sequence by BLAST (available from http://www.ncbi.nlm.nih.gov/blast/) search. The sequence showed 99.9% identity to a BDV sequence derived from a horse (GenBank accession no. DQ251042), which died of BD near the location where the shrews were trapped, and to a BDV sequence from another horse from this region (GenBank accession no. AY374547), which differed by only 1 nt and 2 nt, respectively, confirming the identity of BDVs in this area.

## Conclusions

Bicolored white-toothed shrews (*Crocidura leucodon*) are insectivores that are distributed from central Europe eastward to the Caspian Sea. In Switzerland, these shrews are found in the same areas where BD is endemic in horses, sheep, and other animal species. Bicolored white-toothed shrews are 93–125 mm long and weigh 7–13 g. In northern regions, they often live in gardens, outhouses, and farm buildings, and in the Alps, they can be found <1,600 m above sea level. Shrews are carnivores, and their diet consists of insects and snails, but they may also eat forage (*13*). They live on the ground and do not climb, which could explain why mainly horses (during the grazing season) or animals located in old-fashioned farmhouses without feeding troughs are affected by BD. BD shows an increased incidence in spring and (early) summer, when most shrews are found in pastures and in close contact with grazing animals. In winter, some species of the genus *Crocidura* show social acceptance and group together, which may be a possible source of infection between shrews (*13*). In an experimental study by Sauder and Staeheli (*14*), rats persistently infected with BDV and naive rats were put together, which led to infection of the naive rats after cohabitation. Most animals showed signs of disease 5–6 weeks after first contact with carrier rats, and high virus titers were found in their urine.

In the case of the shrews, no other organs, urine, or body fluids were available. Nevertheless, the experimental results in rats led to the conclusion that persistently infected rodents, in this case, shrews, most likely transmit the virus by excretions such as saliva or urine deposited on forage for horses and sheep. This suggestion is supported by the observation that the bulbus olfactorius near the ethmoid is heavily inflamed in cases of naturally occurring BD. Levels of viral antigen or viral RNA are also high in this region of the brain.

In conclusion, we postulate that shrews are reservoir hosts of BDV. Shrews also meet the criteria established for potential BDV reservoir species in a recent review article (*9*). Our finding, however, does not exclude the possibility that other animal species living in this environment could also harbor BDV. In further studies, we will examine more shrews and additional organs, excretions, and secretions as well as other possible reservoir and vector species.
